# Internet Rumors During the COVID-19 Pandemic: Dynamics of Topics and Public Psychologies

**DOI:** 10.3389/fpubh.2021.788848

**Published:** 2021-12-20

**Authors:** Quan Xiao, Weiling Huang, Xing Zhang, Shanshan Wan, Xia Li

**Affiliations:** ^1^School of Information Management, Jiangxi University of Finance and Economics, Nanchang, China; ^2^School of Management, Wuhan Textile University, Wuhan, China

**Keywords:** COVID-19 pandemic, internet rumors, public psychologies, latent dirichlet allocation, linguistic inquiry and word count, topic modeling

## Abstract

The capturing of social opinions, especially rumors, is a crucial issue in digital public health. With the outbreak of the COVID-19 pandemic, the discussions of related topics have increased exponentially in social media, with a large number of rumors on the Internet, which highly impede the harmony and sustainable development of society. As human health has never suffered a threat of this magnitude since the Internet era, past studies have lacked in-depth analysis of rumors regarding such a globally sweeping pandemic. This text-based analysis explores the dynamic features of Internet rumors during the COVID-19 pandemic considering the progress of the pandemic as time-series. Specifically, a Latent Dirichlet Allocation (LDA) model is used to extract rumor topics that spread widely during the pandemic, and the extracted six rumor topics, i.e., “Human Immunity,” “Technology R&D,” “Virus Protection,” “People's Livelihood,” “Virus Spreading,” and “Psychosomatic Health” are found to show a certain degree of concentrated distribution at different stages of the pandemic. Linguistic Inquiry and Word Count (LIWC) is used to statistically test the psychosocial dynamics reflected in the rumor texts, and the results show differences in psychosocial characteristics of rumors at different stages of the pandemic progression. There are also differences in the indicators of psychosocial characteristics between truth and disinformation. Our results reveal which topics of rumors and which psychosocial characteristics are more likely to spread at each stage of progress of the pandemic. The findings contribute to a comprehensive understanding of the changing public opinions and psychological dynamics during the pandemic, and also provide reference for public opinion responses to major public health emergencies that may arise in the future.

## Introduction

The monitoring and analysis of public opinions is an important task for modern society, as they may provide critical information that enables the timely capture of unanticipated public hotspots and trends, thus contributing to government business continuity (1). Along with the global ravages of the new coronavirus during the COVID-19 pandemic (2, 3), the resulting information explosion poses an unprecedented challenge to public governance (4). Failure to respond to the information effectively would have negative consequences for public health (5).

As the virus spread, rumors were also spread worldwide due to the complex causes, highly specialized prevention & control processes, and strict quarantine measures of the pandemic (6, 7). According to the feature of rumor's generation, the less transparent an event is, the more likely it is to be a hotspot (8). Out of the public's fear of the unknown, Internet rumors have been emerging in various aspects including viruses, health, and livelihoods: from “*drinking soda to prevent new coronaviruses*,” to “*China banning the export of face masks*,” to “*playing in the snow to catch new coronaviruses*” (9). These rumors, a large proportion of which may be misinformation, exacerbated the public's physiological over-reaction to the unknown viruses and their psychological fears (10, 11). Besides the public health impact, the psychosocial offshoots have also been significant (12).

The interconnection provided by the Internet enabled misinformation during the pandemic to have a greater impact on public health (13–16). As the COVID-19 pandemic has gone through approximately one annual cycle, it is informative to conduct a comprehensive analysis on widespread Internet rumors. Such rumors have been spread around since the outbreak of the pandemic, and to clarify the characteristics of the rumors at different stages during the outbreak and circulation of the pandemic would help and provide understanding of public opinion responses to major public health emergencies (1).

Scholars have conducted empirical studies on the intentions and behaviors of Internet users to spread rumors during the pandemic from psychological or behavioral perspectives. For instance, based on the evidence presented in two studies, Pennycook et al. (17) revealed that people shared false claims about COVID-19 partly because they simply failed to think sufficiently about whether the content was accurate when deciding what to share. It has also been found that the spread of rumors about COVID-19 was related to factors such as the age of the spreader, his/her level of education, income, and level of psychological distress (18). As a matter of fact, there is also a close relationship between the spread of Internet rumors and the content of the texts they contain (5, 19). Taking the text of rumors as the object of research can more objectively reflect the distribution, chronological changes, and psychosocial characteristics of Internet rumors during the pandemic than self-reported surveys and experimental studies. In this background, the current study addresses the following research questions (RQs):

***RQ1*** *: During the COVID-19 pandemic, what types of Internet rumors were widely circulated?*
***RQ2*** *: How have public concerns presented by the topic of Internet rumors changed across different stages of the pandemic?*
***RQ3*** *: What kind of psychosocial characteristics were implied in these Internet rumors and how did they differ during different stages as the pandemic evolved?*

Compared to prior studies, the main contributions of this work are three-fold. First of all, distinguishing from the research on the process of participation and spread of Internet rumors, and those studies on the subjective psychological aspects related to rumors, this study takes Internet rumor texts generated during the COVID-19 pandemic as the research target, and analyzes their chronological variation in the distribution of topic types and numbers. Secondly, we reveal the psychosocial dynamics underlying Internet rumors about the pandemic, and through textual linguistic feature analysis, we capture important psychological keywords at different stages, which helps to describe the psychosocial trends and public opinion directions during the pandemic. Thirdly, as an extension of the application of natural language processing (NLP) and text mining techniques in the context of the COVID-19 pandemic, this study sheds light on the significance of utilizing textual data such as news and comments to capture public opinions in a timely and accurate manner, which also provide methodological references for governments or organizations to respond quickly to public health emergencies regarding rumors.

## Related Works

Monitoring public opinions is an essential subject for modern society, especially for widely circulated rumors, which need the extra attention of governments to eliminate public misunderstanding and prevent further social conflicts in a timely manner (1). The outbreak of the COVID-19 pandemic led to a number of Internet rumors, which were characterized by a large number of high density, rapidly spreading retweets and widespread impact (20–23). Online information in emergencies has received a considerable amount of attention from the public in recent years (5). As the COVID-19 pandemic has caused unprecedented panic and concern around the world, the scale and speed of information diffusion also increased dramatically since the outbreak (9). The endless rumors have brought more anxiety and distress in society than just the virus, and have inflicted great damage on people's enthusiasm to fight the pandemic (24). Therefore, it is essential to conduct targeted research into the Internet rumors that have been generated during this period (1, 25), which will facilitate a better understanding of the real trends of COVID-19, avoid unnecessary anxiety, and restore normal social order and stability of society (26).

As a matter of fact, there has been long-term research on Internet rumors. Scholars have been focusing on the definition of Internet rumors (15), motivation for rumor sharing (21), spreading models and veracity of rumors (27, 28), refutation of rumors (29), determining whether Internet rumors are true or false (30), and policy on the governance of Internet rumors (21), etc. The methods that have been proposed in the past for the study of Internet rumors are of value for investigating the Internet rumors during the COVID-19 pandemic. For example, in terms of the intention to share rumors about the pandemic, researchers noted that people were more likely to focus on issues that affected their personal interests based on online rumors on Twitter, thus those who were affected more by the pandemic were more likely to discuss and share related information (31, 32). Regarding the influence of online rumors about the pandemic, several studies have shown that rumors about health, virus-related, and therapy-related topics were not only detrimental to public health but also continued to influence individual behavior (17, 32). In addition, in studies on the governance of COVID-19-related Internet rumors, it was suggested that researchers should pay attention to their risks and hazards, and the government should take timely regulatory measures (18, 33, 34).

Rumors usually take the form of text and spread rapidly among the population through social media, instant messaging, websites, etc. Therefore, the analysis of the textual content of rumors is essential for COVID-19 public opinion research (5). In terms of textual analysis of rumors, Samia et al. (34) argued that advanced technologies such as NLP or data mining could be used to detect and remove online content about COVID-19 that lack a scientific basis on social media platforms. In relation to the propagation characteristics of pandemic rumors, the research of Singh et al. (14) showed that COVID-19-informed users tended to use more narratives than misinformed users. Zhu et al. (1) proposed an early warning scheme considering the multiple factors of Internet public opinion and the dynamic characteristics of burst events. Zeng et al. (5) proposed an approach for fake news detection by comprehensively mining the semantic correlations between text content and attached images.

However, in general, in-depth research into the text content of Internet rumors about the COVID-19 pandemic is far from adequate compared to traditional Internet rumors issues. We address text analysis and its mining as an effective way to gain understanding of rumors during the pandemic. On one hand, in the Internet environment, textual data analysis techniques are in line with the 4V characteristics of big data (i.e., Volume, Variety, Value, Velocity), and by parsing the grammatical and semantic structure of the rumor texts, it can help to understand the distribution of topics throughout pandemic progression. On the other hand, by combining statistical analysis techniques with deep excavation techniques for the text of Internet rumors during the COVID-19 pandemic, the psychological dynamics and behavioral characteristics underlying the text can be captured more comprehensively, objectively, and concretely than traditional statistical analysis based solely on numerical data and individual self-report-based surveys.

## Method

**Figure 1:**
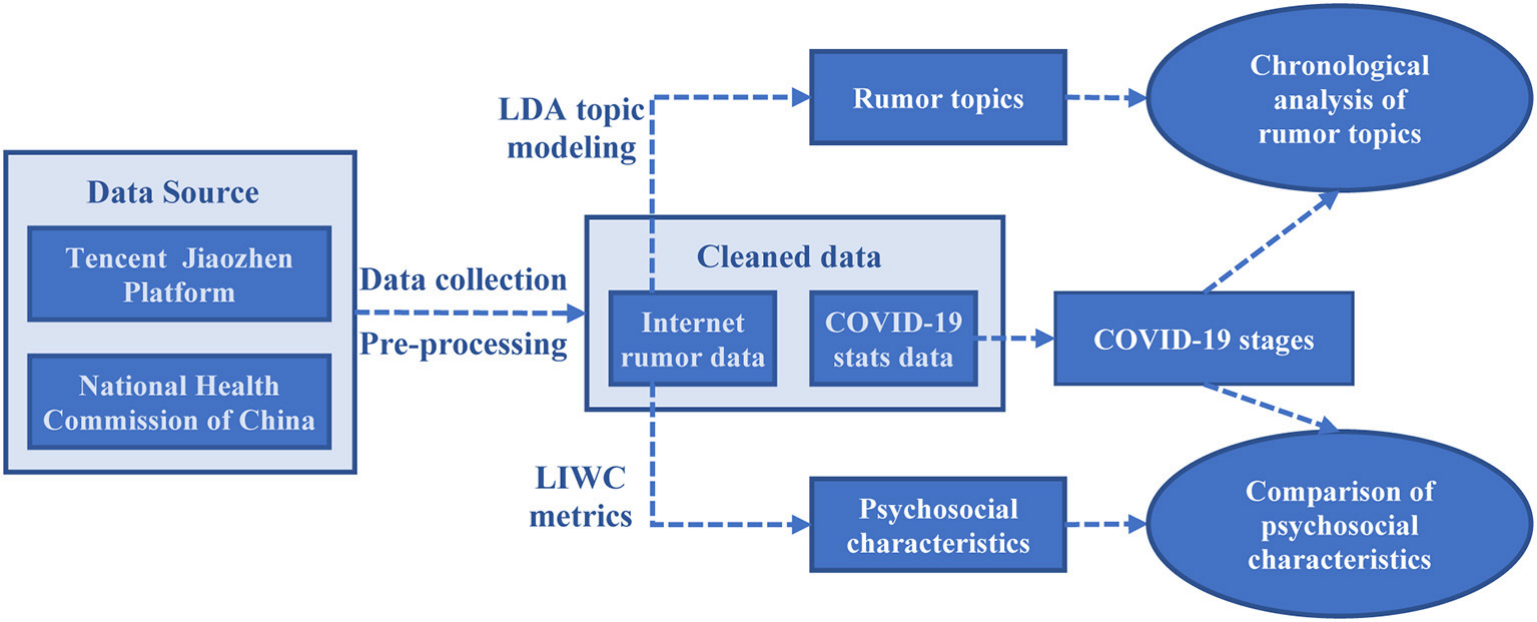
Research framework.

The research framework of this study is illustrated in Figure 1. Firstly, web crawlers are deployed to crawl Internet rumor data and COVID-19 pandemic statistics from Tencent's “JiaoZhen” platform and the website of the National Health Commission of China. After pre-processing the crawled data, a cleaned dataset is obtained for the subsequent steps of analysis. Then, the popular rumor topics are mined by performing LDA topic modeling, and the psychosocial characteristics inherent in the rumors are captured based on the LIWC metrics. After modeling the COVID-19 evolving stages through the COVID-19 pandemic stats data, two sub-studies, i.e., chronological analysis of rumor topics and comparison of psychosocial characteristics, are conducted. The detailed elaboration of the research methodology is given below.

### Data Collection and Pre-processing

We choose Tencent's “JiaoZhen” platform as the data source of Internet rumors for our study because it is a scientific rumor verification platform built by the China Medical Doctors Association and the largest social platform in China, Tencent^1^ Rumors that are widely spread on the Internet would be released in a short period of time on this platform for the public to identify. The rumor data on the platform contain information such as rumor title, summary, key points of verification, the original text of rumor, tag of rumor, date of verification, etc. More importantly, the “JiaoZhen” platform provides authoritative verification of whether the widely circulated rumors are true (truth) or false (misinformation).

**Figure 2:**
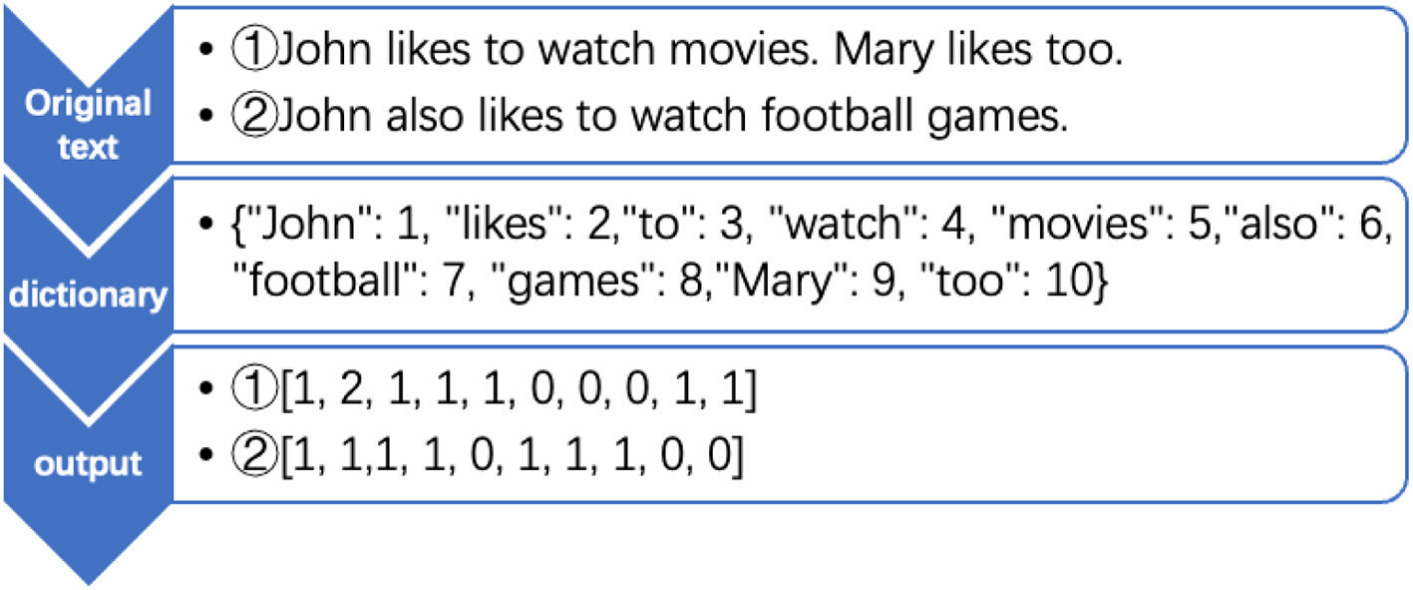
Word2Vec model.

As the crawled raw data are informative and the relationships between data items are vague, pre-processing is needed. Data pre-processing involves three tasks: The first task is the elimination of incomplete rumor cases, which is required if a data record misses critical information on key variables. Secondly, the full text of each rumor is separated into a words list based on a customized dictionary. As the texts of the pandemic rumors contain a large number of medical and biological terms, which are mostly in Chinese, it may lead to difficulties and ambiguities in the processing of the corresponding English professional terms, so it is necessary to merge the dictionaries of these two languages. The third task is the quantification of textual data. Data in text form cannot be directly calculated, so the textual dataset needs to be converted into numerical matrices. Commonly used conversion methods include Word2Vec and TF-IDF, after a pilot test, Word2Vec is selected for conversion to model and lexicase the natural language information in rumor texts (see Figure 2).

### LDA Topic Modeling

#### Principles of Modeling

The LDA model is a document generation model that involves a Bayesian probability structure with three levels: document, topic, and word. The generation process utilizes the Word2Vec model, and it is assumed that a document is composed by selecting topics with a certain probability, and these topics in turn select feature words with a certain probability. The LDA model, as an unsupervised machine learning technique, adopts the inverse procedure of the above process.

**Figure 3:**
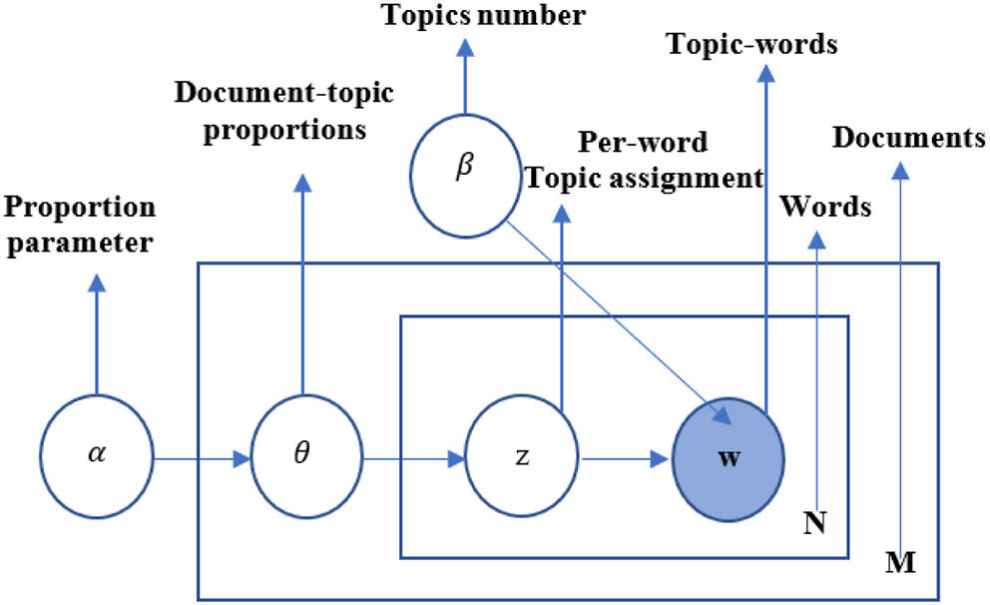
The LDA model.

The probabilistic diagram of the LDA model is shown in Figure 3, where *M* is the number of documents in the document collection and *N* is the number of words contained in the documents. In the LDA model, for a given set of documents, it is assumed that they contain *K* topics. The model generates *K* multinomial distributions based on the Dirichlet distribution with hyperparameter β. Each distribution represents the probability of a random generation of words in the entire lexicon for that topic. The formula is as follows:


$$\begin{array}{l} \varphi_{k}\sim Dirichlet\left(\beta \right);\;k=1,2,\ldots ,k,\varphi_{k}\in R^{w},\;\beta \in R^{w} \end{array}$$

where *W* is the size of the entire lexicon; β and φ_*k*_ are real number vectors with *W* dimension, and φ_*k*_ is a vector of values corresponding to the probability distribution of the topic *k*. The absolute value of β in each dimension is related to the importance of prior knowledge, and its relative value in each dimension determines the probability of the vocabulary occurring in the dimension.

#### Optimal Number of Topics

##### Calculation of Topic Perplexity

Topic perplexity is a measure of the effectiveness of a probability distribution or probability model in predicting a sample. It can also be used to compare two probability distributions or probability models. In training the LDA model, the value of perplexity generally decreases as the number of topics increases, and the number of topics at a relative low level of perplexity is considered to be acceptable. Perplexity is calculated as:


$$\begin{array}{l} Perp\left(w\right)=2^{H\left(w\right)}={p\left(w_{1}w_{2}\ldots w_{n}\right)}^{-\;\frac{1}{n}}, \end{array}$$

where *p*(*w*_1_*w*_2_…*w*_*n*_) is the document frequency of the word *w*; *H*(*w*) is the entropy of each word which is calculated as:


$$\begin{array}{l} H\left(w_{1},w_{2},\;\ldots ,w_{n}\right)=-\log_{w_{1}^{n}\in L}P\left(w_{1}^{n}\right)\log P\left(w_{1}^{n}\right),\\ \;L=\left\{w_{1}^{n}\;,n=1,2,\;\ldots ,N\right\}. \end{array}$$

##### Calculation of Topic Coherence

Topic coherence is another quantitative metric to evaluate the optimal number of topics for the LDA model. In general, the value of coherence increases as the number of topics increases in LDA training, and the number of topics with a relative high value of coherence is determined as satisfied. Topic coherence typically works with visualization tools of topic data, such as the Python package pyLDAvis, to jointly determine the optimal number of topics. Topic coherence is calculated as:


$$\begin{array}{l} C\left(z;S^{z}\right)=\overset{N}{\underset{n\;=\;2}{\sum }}\overset{n-1}{\underset{l\;=\;1}{\sum }}log\frac{D_{2}\left(w_{n}^{z},w_{l}^{z}\right)+1}{D_{1}\left(w_{l}^{z}\right)} \end{array}$$

where $S^{z}=\left\{w_{1}^{z}\ldots w_{n}^{z}\right\}$ is the set of words in the first N documents; *D*_1_(*w*) is the frequency of documents containing the word w, and *D*_2_(*w*_1_,*w*_2_) is the frequency of documents where the two words *w*_1_, and *w*_2_ co-occur.

### Chronological Analysis of Rumor Topics

This study utilizes daily data of the COVID-19 pandemic in China (released by the National Health Commission of China) as the basis for dividing the stages of pandemic progression^2^ and discriminating the characteristics of the rumor's topics between different stages. The featured points for the cumulative number of cases (rate) and the number of new cases (rate) are used as cut-off points between the different stages. Combined with the result of the LDA model, the chronological characteristics of the Internet rumors during the pandemic are investigated from two perspectives: (i) the changes of the number of rumors for each topic across different stages, and (ii) the composition of each rumor topic within each stage.

### Comparison of Psychosocial Characteristics Between Rumors

LIWC (Linguistic Inquiry and Word Count) is an NLP technology that quantifies the content of text and counts different categories of words in the text, especially psychological words, such as the frequency of causal, emotional, and cognitive words. This method was proposed by Pennebaker et al. (35) during their research into the therapeutic effects of emotion writing, and it is currently being developed as desktop software and programming packages (36). In the twenty years since its introduction, LIWC has been widely adopted by psychological researchers in a variety of areas due to its reliability (37, 38). LIWC's core dictionary divides the lexical attributes and corresponding word lists, and contains 4 general descriptive categories (i.e., *WordCount, WordPerSentence, RateDicCover*, and *RateSixLtrWord*), 22 linguistic categories (e.g., *Pronoun, AuxVerb*, etc.), 32 psychological categories (e.g., *Social, CogMech, Affect*, etc.), 7 personalization categories (e.g., *Work, Leisure, Family*, etc.), 3 paralinguistic categories (e.g., *Assent*, etc.), and 12 punctuation categories, for a total of 101 word categories and around 4,500 words^3^ (38). In this study, relevant word categories are screened according to their topic fitness and then *t*-tests are conducted for the psychosocial characteristics between truth and misinformation, and F-tests are conducted for the psychosocial characteristics of Internet rumors at different stages, respectively.

## Results

A total of 652 COVID-19 rumors are collected from the “JiaoZhen” platform, from the first related rumor titled “Wuhan unexplained pneumonia is SARS virus” on January 18, 2020, to the completion of this study on October 2, 2020. We also obtain daily COVID-19 pandemic situation data for this period from the National Health Commission of China website, which are used to segment the pandemic stages by the number of cumulative and new infections. The results of topic extraction, topic chronological characteristics, and comparison of psychosocial characteristics are as follows.

### Topic Extraction

**Figure 4:**
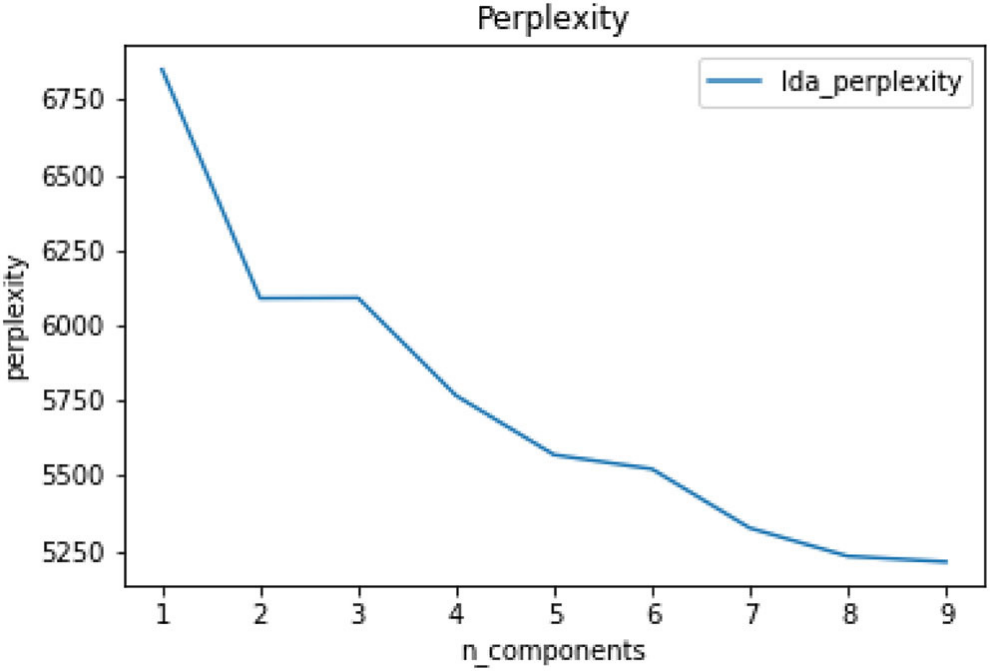
Calculation of perplexity.

**Figure 5:**
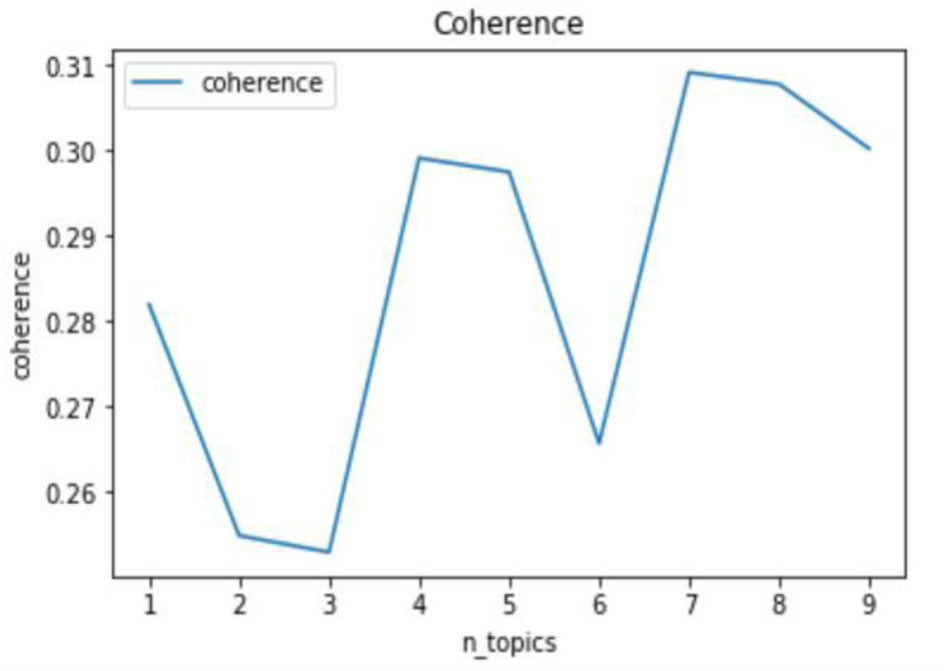
Calculation of coherence.

**Figure 6:**
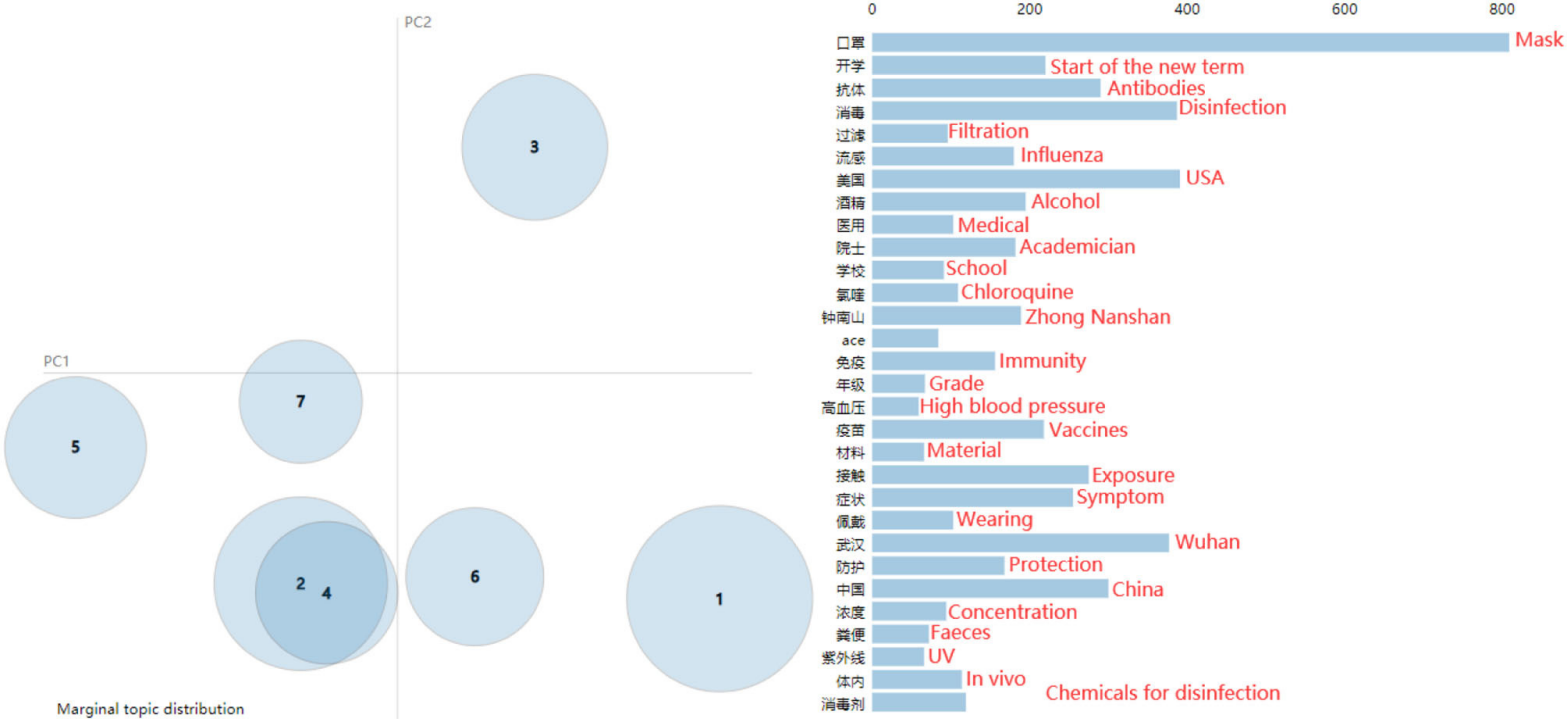
Visualization of topics.

Figures 4, 5 show the results of the topic perplexity and topic coherence for the training of the LDA model, which demonstrate that 7 and 8 could be the optimal number of topics according to the determining criteria by perplexity and coherence. After taking the actual rumor texts into consideration, the preliminary number of topics for the LDA model is set as 7, then we conduct a topic visualization through the Python LDA visualization package, pyLDAvis. As shown in Figure 6, if the number of topics is set to 7, topic 4 is almost covered by topic 2 in the topic visualization graph, so the final number of topics for subsequent analysis is determined to be 6. As high-frequency keywords such as mask, Wuhan, antibodies, and disinfection appear in almost all topics, they are excluded from the dictionary for the subsequent analysis. The six topics extracted, the keywords in each topic, and examples of rumor titles are shown in Table 1. Based on the keywords that appeared in each topic and the corresponding rumor texts, we name the six topics as: “Human Immunity” for topic 1, “Technology R&D” for topic 2/4, “Virus Protection” for topic 3, “People's Livelihood” for topic 5, “Virus Spreading” for topic 6, and “Psychosomatic Health” for topic 7.

**Table 1:** Extracted topics, keywords and examples of rumor texts.

**Topic**	**Topic names**	**Top 8 keywords**	**Examples of rumor texts**
Topic1	Human immunity	Antibodies, Body, Cell, Immunity, *In vivo*, Temperature, Concentration, Feces	“Bird's nest can boost immune system and prevent new coronavirus,” “Resistance can be improved by heavy exercise during the pandemic”
Topic2/4	Technology R&D	Chloroquine, Zhong Nanshan, Gene, Academician, Italy, USA, Sequence, Japan	“Resveratrol may treat and prevent novel coronavirus,” “India has developed a nano spray, a spray object surface 90 days free of viruses”
Topic3	Virus protection	USA, Influenza, Filtration, Protection, Ultraviolet light, Wearing, Materials, Medical	“Do not wear sweaters or clothing jackets with fur collars or fleece, they tend to attract viruses,” “Flower lotion with 70–75% alcohol content can effectively prevent the new coronavirus”
Topic5	People's livelihood	Start of the new term, school, grade, internet, refuting rumors, official, media, social	“During the pandemic, you cannot go out for a walk, as it is easy to be infected,” “2020 National College Entrance Examination postponed for one month”
Topic6	Virus spreading	Exposure, patients, droplets, disease, seafood, south in china, crowd, air	“Pangolin as an intermediate host for the novel coronavirus,” “Willow flock can carry new coronaviruses, leading to trans-regional spreading”
Topic7	Psychosomatic health	Isolation, high blood pressure, blood vessels, heart, antihypertensive drugs, exercise, injury, nervous	“Antihypertensive drugs increase the risk of viral infections in patients with high blood pressure,” “Children wearing N95 masks may cause irreversible damage”

### Topic Chronological Characteristics

**Figure 7:**
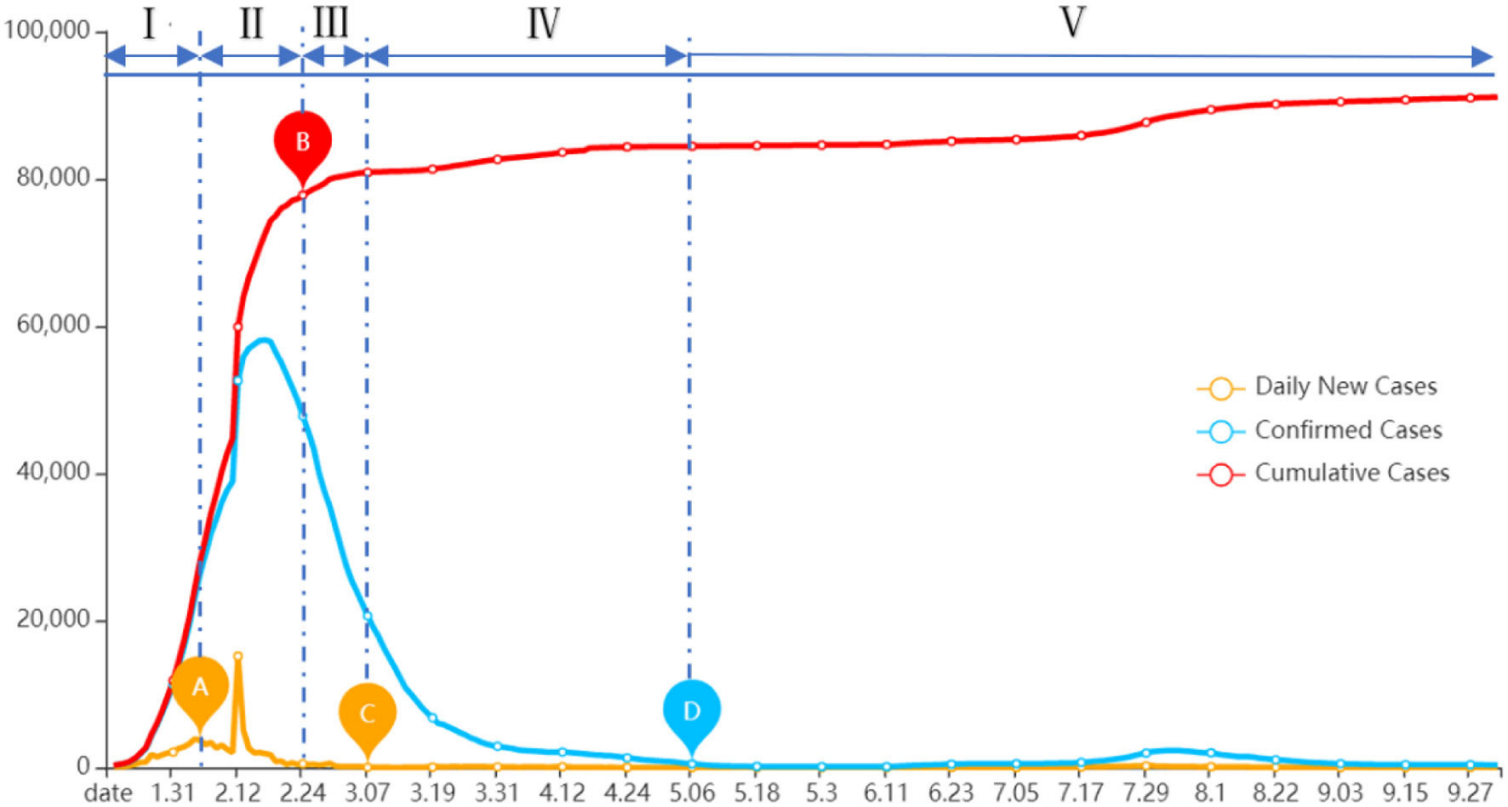
Data on progress of the COVID-19 pandemic in China.

**Figure 8:**
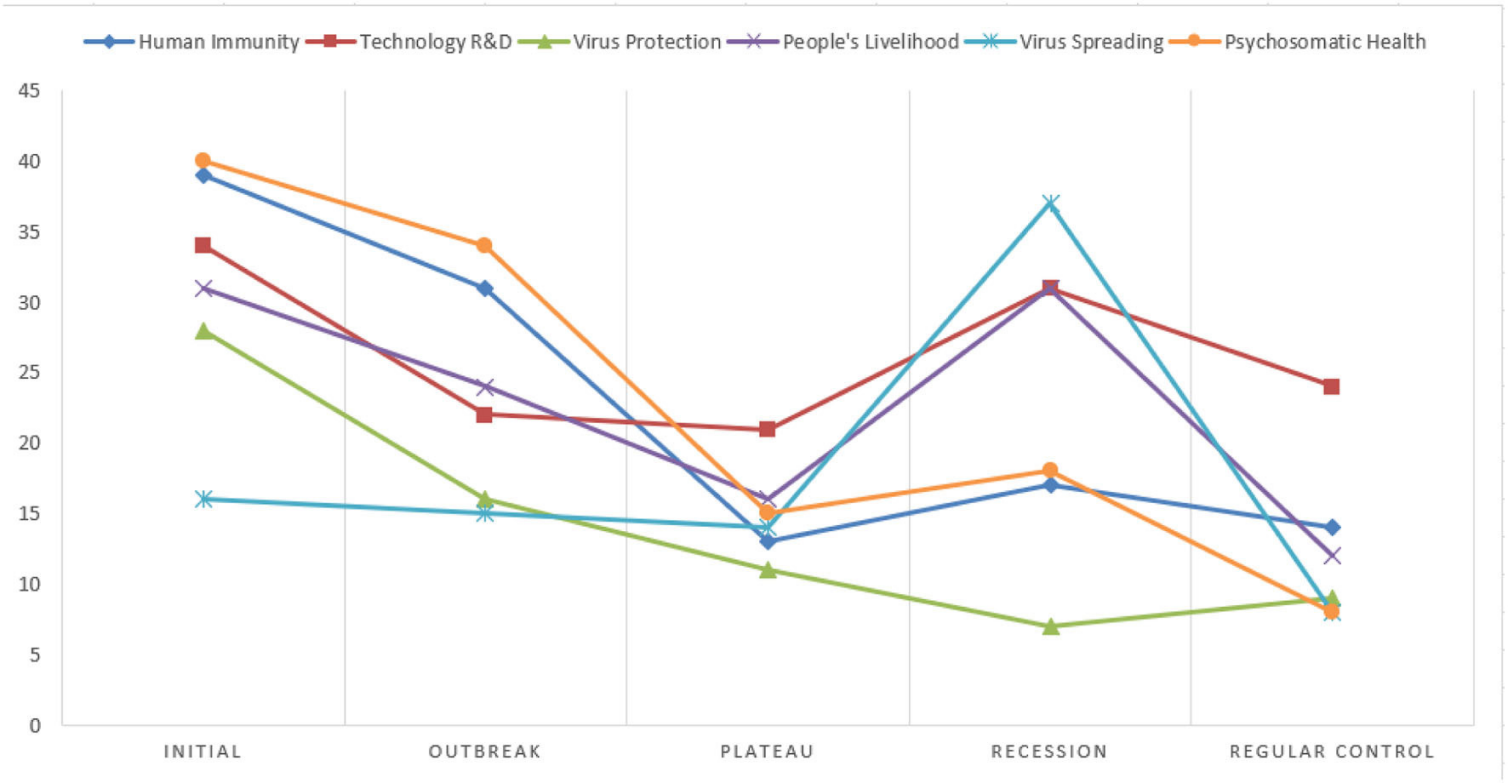
Dynamics in the number of rumors on different topics.

We obtain the daily disclosure of the COVID-19 pandemic situation from January 18 to October 2, 2020, from the official website of the National Health Commission of China. As shown in Figure 7, the red curve depicts the number of confirmed cases added daily, the blue curve shows the number of uncured confirmed cases per day, and the orange curve indicates the number of cumulative cases. During the progression of the pandemic, we identify four key time points. The first time point is when the daily new cases reach a local maximum (point A), the second key time point is when the cumulative cases start to level off (point B), the third point is when the new cases approach zero (point C), and the last one is when the confirmed cases approach zero (point D). Based on the four key time points for segmentation, the life cycle of the pandemic progress is divided into the following five stages: (I) Initial stage (January 18–February 5), (II) outbreak stage (February 6–February 21), (III) plateau stage (February 22–March 8), (IV) recession stage (March 9–May 7), and (V) regular control stage (May 8–October 2). Based on this division of the stages of the pandemic, the chronological characteristics of Internet rumors during the pandemic, in terms of the changes of the number of rumors for each topic across different stages, and the composition of each rumor topic within each stage are investigated and the result is shown in Figure 8.

Regarding the number of rumors for each topic across stages, rumors in “Human Immunity” and “Psychosomatic Health” topics share a very similar trend over time, with the highest numbers in the early stages of the pandemic and followed by a gradual decline and then fluctuations. Rumors in the topics of “Technology R&D,” “People's Livelihood,” and “Virus Spreading” are on a steady decline in the first three stages, but rebound rather sharply in the recession stage of the pandemic, while the number of “Virus Protection” rumors has continued to decrease slowly over time. As far as the composition of each rumor topic within each stage is concerned, in Figure 8 it can be found that at the initial stage and outbreak stage of the pandemic, “Human Immunity” and “Psychosomatic Health” account for a greater proportion of rumors. During the plateau stage, the number of Internet rumors declines for all topics, with a higher number within the “Virus Protection” topic. During the recession stage, the number of rumors for all topics rebounds, with the highest number of rumors for the “Virus Spreading” and “People's Livelihood” topics. During the regular control stage, the number of Internet rumors by and large is down to the lowest, with the highest number in the “Virus Protection” category of topics.

### Comparison of Psychosocial Characteristics

#### Comparison Between Truth and Misinformation

Tencent's “JiaoZhen” platform provides a label for each Internet rumor to mark it as truth or misinformation. These labels are certified by authoritative experts or institutions and are accompanied by corresponding evidence for argumentation. Based on the LIWC software package, we conduct a *t*-test on the psychosocial characteristics between truth and misinformation. Among all characteristics, 24 are significantly different. The results are shown in Table 2. The full name, category, and word examples of the identified characteristics are provided in Table A1 in the Appendix.

**Table 2:** *T*-test results for truth and misinformation.

**Categories**		***T*** **-test for equality of means**			**Categories**		***T*** **-test for equality of means**		
		***T* value**	***p***	**MD**			**T value**	***p***	**MD**
Psychological characteristics	Social	−3.210	0.001	−0.011	Linguistic characteristics	Verb	2.845	0.005	0.014
	Friend	−2.138	0.033	−0.001		AuxVerb	3.384	0.001	0.010
	Affect	2.418	0.016	0.008		Number	−3.316	0.001	−0.005
	CogMech	3.804	0.000	0.024		Interjunction	2.282	0.023	0.008
	Cause	2.524	0.012	0.005		TenseM	−2.804	0.005	−0.007
	Discrep	3.044	0.002	0.009		ProgM	−2.753	0.006	−0.004
	Tentat	2.203	0.028	0.005	Personal	Relative	−2.977	0.003	−0.020
	Inclusive	3.436	0.001	0.009		Space	−4.085	0.000	−0.023
	See	−2.183	0.029	−0.002		Leisure	−4.559	0.000	−0.012
	Bio	2.885	0.004	0.019	General Description	Qmark	5.778	0.000	0.007
	Health	5.764	0.000	0.030		WordPerSentence	−2.347	0.019	−1.719
	Ingest	−3.303	0.001	−0.011		RateSixLtrWord	2.360	0.019	0.002

*MD stands for Mean-difference, where a positive value indicates that the characteristics in truth has a higher value than in misinformation, while a negative value indicates the opposite. Considering the limited space, insignificant characteristics are not listed. The meanings of each characteristics can be referred to Tausczik and Pennebaker (39), and Table A1 in Appendix*.

In terms of the psychological characteristics, the means of truth are significantly higher than misinformation (*p* <0.05) for *Affect, CogMech, Cause, Discrep, Tentat, Inclusive, Bio*, and *Health*, whereas the means of psychological characteristics of *Social, Friend, See*, and *Ingest* in misinformation are significantly higher than in truth. Significant differences are also found in the linguistic characteristics, where the truthful text contains more *Verb, AuxVerb*, and *Interjunction*, while misinformation contains more *Number, TenseM*, and *ProgM*. Among the personalized characteristics, the two also differ markedly. The percentage of *Relative, Space*, and *Leisure* words in truth are significantly less than these in misinformation. In the general description characteristics, the average number of words per sentence (*WordPerSentence*) differs significantly between the two categories of rumors with a mean-difference of −1.719, demonstrating that misinformation has a considerably higher number of words than true statements. The characteristics of *Omark* and *RateSixLtrWord* are higher in truth than misinformation.

#### Comparison Across Different Stages

In order to capture the differences of psychosocial characteristics of rumors at different stages, an F-test is conducted, and the characteristics that significantly differ across stages (*p* <0.05) are shown in Table 3. From the *F*-test results it can be observed that for the psychosocial characteristic of *CogMech*, its frequency is significantly lower in the recession stage than in the other four stages (mean-difference <0), while *Insight* words are significantly higher in the regular control stage than in the other four stages (mean-difference >0). In terms of the *Bio* and *Health* characteristics, the mean values of the plateau stage, recession stage, and regular control stage are significantly lower (mean-difference <0) than those of the initial and outbreak stages, and their mean differences varied significantly. Among the *Personal* category of psychosocial characteristics, *Space, Time, Work*, and *Religion* have significant differences across stages. *Space* has significantly lower mean values in the outbreak stage than in the next stages, *Work* is significantly higher in the recession stage than in the other four stages, while *Religion* has significantly higher mean values in the regular control stage than in the previous four stages (*p* <0.001), but the MD values are not large (MD ≤ 0.006). In general, we have found that the widely circulated Internet rumors during the COVID-19 pandemic exhibited rather noticeable differences in psychosocial characteristics at different stages of pandemic progression.

**Table 3:** *F*-test results for rumors across different stages.

**V**	**I**	**J**	**MD (I-J)**	***p***	**V**	**I**	**J**	**MD(I-J)**	***p***
CogMech	4	1	−0.030	0.000	Health	3	1	−0.019	0.012
		2	−0.041	0.000			4	0.017	0.033
		3	−0.025	0.010		4	1	−0.035	0.000
		5	−0.031	0.002			2	−0.028	0.000
Insight	5	1	0.010	0.000			3	−0.017	0.033
		2	0.009	0.003		5	1	−0.033	0.000
		3	0.009	0.005			2	−0.026	0.002
		4	0.008	0.012	Space	2	4	−0.022	0.005
Discrep	1	2	−0.006	0.100			5	−0.036	0.000
		3	0.010	0.015		5	1	0.023	0.010
		4	0.019	0.000			2	0.036	0.000
		5	0.015	0.001	Time	4	1	0.017	0.000
	2	1	0.006	0.100		5	1	0.022	0.000
		3	0.016	0.000			2	0.014	0.011
		4	0.025	0.000			3	0.014	0.027
		5	0.021	0.000	Work	1	3	−0.014	0.049
	3	1	−0.010	0.015			4	−0.045	0.000
		2	−0.016	0.000			5	−0.017	0.026
		4	0.009	0.033		4	1	0.045	0.000
Bio	3	1	−0.025	0.010			2	0.035	0.000
		2	−0.020	0.047			3	0.031	0.000
		4	0.027	0.007			5	0.028	0.000
	4	1	−0.052	0.000	Religion	5	1	0.005	0.000
		2	−0.047	0.000			2	0.006	0.000
		3	−0.027	0.007			3	0.006	0.000
	5	1	−0.044	0.000			4	0.005	0.000
		2	−0.039	0.000					

*I and J represent the two stages compared, where 1 for Initial stage (Jan 18–Feb 5), 2-Outbreak stage (Feb 6–Feb 21), 3-Plateau stage (Feb 22–Mar 8), 4-Recession stage (Mar 9-May7), 5-Regular Control stage (May 8–Oct 2). MD(I-J) is the mean-difference between I and J phases, where a positive value means the value of I stage is larger than J stage, and a negative value is the opposite*.

## Discussion and Implication

### Discussion

By summarizing the results presented above, we obtain three major findings. Firstly, we have found that in the face of a serious unknown public health emergency such as the COVID-19 pandemic, personal health is a concern of the highest priority in the early stages, which is consistent with the results of the survey by Xiong et al. (40) and Apuke and Omar (41). Our study further supports this assertion in terms of the distribution of rumor topics and the dynamic of the psychosocial characteristics. During the initial stage and outbreak stage of the COVID-19 pandemic, the rumor topics are highly distributed in the categories of “Human Immunity” and “Psychosomatic Health,” which are closely related to personal health. Among our extracted six topics (i.e., “Human Immunity,” “Technology R&D,” “Virus Protection,” “People's Livelihood,” “Virus Spreading,” and “Psychosomatic Health”), “Human Immunity” accounts for the largest number of Internet rumors, reflecting the fact that there are still large unknowns and gaps in human immunity, and that the more unknowns there are, the more attention they get. In terms of text semantics, the mean values of *CogMech* and *Health* are significantly lower in the plateau stage, the recession stage, and the regular control stage than these in the initial stage and outbreak stage (MD <0). It is noteworthy that the results of the *t*-test of psychological characteristics words show that true statements contain more personal emotion as well as more expressions of real feelings, where especially the Internet rumors containing more *CogMech* (*Health* and *Ingest*) would generate greater credibility.

Secondly, the study reveals that as the pandemic progressed, public concerns switched from individual-centered to public-centered. From the chart of dynamics of rumor topics (Figure 8), it can be found that during the pandemic, there is a dynamic shift from a large percentage of personal-related rumors to more social concerns, from “Human Immunity” and “Psychosomatic Health” at the top to “Technology R&D” and “People's Livelihood” dominating during the pandemic. The topics of “Human Immunity” and “Psychosomatic Health” top the list during the initial and outbreak stages, with greater attention and influence in the spread of public opinion. While during the plateau stage, the six categories of topics are almost evenly distributed and the public begins to distract their attention to a certain extent, pointing out that people have somewhat accepted the negative impact brought about by the outbreak of the COVID-19 pandemic and developed more comprehensive considerations and concerns. The dynamic analysis of the psychosocial semantics of rumor texts confirms this finding. The significant change in the mean value of the *Personal* category of psychosocial characteristics indicates that as the pandemic situation improves, the general public's attention moved to socio-economic, government welfare, school opening, and other livelihood issues. The switch in concern to public issues suggests that the public “side effects” of the pandemic (such as the decline in economic indicators) are beginning to take effect after hitting individual health.

Thirdly, the psychology of the whole community has changed from “fear” to “worry,” with an overall positive trend. During the initial stage, people's fear of unknown viruses is at its peak, and the most attention is paid to Internet rumors about “Human Immunity” and “Psychosomatic Health.” After the plateau stage, the pandemic tends to calm down, people pay more attention to livelihood and public policies, etc. Although the number of Internet rumors decreases, the dissemination is still at a high level and the textual emotions are mainly rendered as worry. The significant change of *CogMech* at different stages indicates that after three stages of fighting against the new coronavirus, i.e., the initial stage, the outbreak stage, and the plateau stage, people have switched from not knowing anything to being familiar with it, reflecting to a certain extent the accepting of reality of the pandemic by the general public. Specifically, the *Insight* word belonging to the *CogMech* category has changed the most, which is due to the fluctuation of the pandemic during the regular control stage and how such new coronaviruses are being redefined and re-perceived (37, 38, 42). It is this process of introspection that allows individuals to reinterpret the pandemic and improve their health. In the meanwhile, the words in terms of *Discrep*, which also belongs to the *CogMech* category, continues to increase, suggesting that human knowledge of the viruses and the development of corresponding technologies is in a constant state of progress. All in all, the overall cognitive characteristics show a gradual improvement in the psychological state of the public.

### Implication

Our study generates contributions to the literature on the COVID-19 pandemic, Internet rumor, and to related management practices. Prior studies on Internet rumors during the COVID-19 pandemic have addressed its hazards to public health along with the necessity and urgency of its research regarding the spreading characteristics (43, 44), while some studies indicated that inauthentic articles or misinformation on social media platforms were more likely to be forwarded and shared than those that were reliable (45–47). However, the regularity of these widely circulated rumors changing in topic over time and the implied psychosocial characteristics of the public have not received sufficient attention. After earlier opinion-based or commentary explorations (41), some studies have tried to find the behavioral intention of the public to disseminate information during the pandemic as well as antecedents through questionnaires or laboratory experiments (17, 40). However, such cross-sectional and self-reported data sources not only lack objectivity, but also the generality of the results is limited by the sample and, in addition, cannot give a concrete and dynamic understanding of the psychological changes of the public. Going a step further, this study drills down into the textual content of Internet rumors to systematically present the changing topic distribution of Internet rumors with pandemic progression and the implied psychological characteristics at different stages. We extend the techniques of natural language processing and text mining in computing areas to the COVID-19 pandemic and public psychometrics to fill the gaps in existing research, and provide ideas for more generalized, real-time, big data-driven research in the field that utilizes objective data in the future.

The explosion of Internet rumors during the COVID-19 outbreak did make it extremely difficult for the government to grasp public opinion and for the public to recognize the truth of information. Nevertheless, our study provides ideas for responding to public health emergencies. The change in the volume of Internet rumors during the stages of the pandemic suggests that at the beginning of a public health emergency when Internet rumors are exploding, it is effective and efficient to devote more resources to timely verification of rumors and to deliver and spread correct information to the public to avoid panic. Since the public is initially concerned about personal health, while often the health effects of new viruses are unknown, it is necessary for the government to establish close ties with professional medical institutions to provide the public with professional advice in the first instance, and to offer them the expertise to protect their personal health. Due to the dynamic nature of pandemic progression, the government should pay attention to the changes in public opinion that may be brought about by each rebound of the pandemic. For example, to provide the public with information on the characteristics of newly mutated strains and prevention strategies, and to release them to the public in advance before misinformation is widely disseminated. It is essential to establish an official channel for rumor verification and timely information disclosure. The general public should be aware that Internet rumors do increase dramatically with a pandemic outbreak, and should have a certain ability to identify rumors, especially in the early stages, to avoid getting lost in the flood of personal health rumors. In the event of a rebound of each outbreak, or a new public health emergency in the future, it is recommended to follow professional medical advice and not to forward or share rumors to others without being certain of their truth or falsity.

## Conclusion

The outbreak of a severe pandemic such as COVID-19 is bound to bring about personal physiological sensitivity and psychological panic, and the resulting Internet rumors generate more psychological panic and social impact on the public due to the contagion effect of the Internet. We argue that the textual content of Internet rumors is an important source for tapping into the public psyche and understanding the social impact of severe epidemics. In this paper, we investigate the spreading and textual characteristics of Internet rumors during the COVID-19 pandemic by utilizing the LDA model and LIWC metrics. Theoretically, this study provides a systematic and objective view of the chronological changes in the topics of such distinctive and densely generated Internet rumors as well as the significant changes in public psychology in the context. While in practice, the methodology and findings of this study can serve as a reference for the government and health organizations to understand the public opinion situation and prevent the spread of misinformation (20, 48).

As an exploratory study of this unprecedented pandemic, the current study also has some limitations. For example, the data for the study are obtained from a rumor verification platform, where the information disclosure has a certain delay in time. The rumors and pandemic data are from China, thus lacking empirical evidence on a global scale. To this end, future research can obtain a larger amount of heterogeneous data related to rumors from social media and from around the world, utilize big data analytics for more in-depth analysis, and capture public opinions more quickly and accurately by building rumor identification and public opinion management platforms, so as to respond to sudden public health crises that may occur again.

## Data Availability Statement

The datasets presented in this study can be found in online repositories. The names of the repository/repositories and accession number(s) can be found in the article/supplementary material.

## Author Contributions

QX: conceptualization, methodology, and writing—reviewing and editing. WH: writing—original draft preparation. XZ: conceptualization and validation. SW: methodology and data curation. XL: validation. All authors contributed to the article and approved the submitted version.

## Funding

This work was supported by the National Natural Science Foundation of China under grants 71861014, 71863015, and 71974152, the China Postdoctoral Science Foundation under grant 2019M652272, the China Social Science Foundation under grant 20ZDA047, Priority Postdoctoral Research Projects of Jiangxi Province under grant 2018KY10, the Science and Technology Project of Jiangxi Education Department under grant no. GJJ60458, and the Social Science Project of Jiangxi Province under grant 17BJ31.

## Conflict of Interest

The authors declare that the research was conducted in the absence of any commercial or financial relationships that could be construed as a potential conflict of interest.

## Publisher's Note

All claims expressed in this article are solely those of the authors and do not necessarily represent those of their affiliated organizations, or those of the publisher, the editors and the reviewers. Any product that may be evaluated in this article, or claim that may be made by its manufacturer, is not guaranteed or endorsed by the publisher.

## Figures and Tables

**Table A1:** Full name, category and word examples of the identified characteristics.

**Abbreviation**	**Full title**	**Category**	**Examples**
Affect	Affective process	Psychological characteristics	happy, cried, abandon
AuxVerb	Auxiliary verbs	Linguistic characteristics	am, will, have
Bio	Biological process	Psychological characteristics	eat, blood, pain
Cause	Causation	Psychological characteristics	because, effect, hence
CogMech	Cognitive process	Psychological characteristics	cause, know, ought
Discrep	Discrepancy	Psychological characteristics	should, would, could
Friend	Friend	Psychological characteristics	buddy, friend, neighbor
Health	Health	Psychological characteristics	clinic, flu, pill
Inclusive	Inclusive	Psychological characteristics	and, with, include
Ingest	Ingestion	Psychological characteristics	dish, eat, pizza
Insight	Insight	Psychological characteristics	think, know, consider
Interjunction	Interjunction	Linguistic characteristics	and, but, whereas
Leisure	Leisure	Personal	cook, chat, movie, apartment, kitchen,
Number	Number	Linguistic characteristics	second, thousand
ProgM	Progressive marks	Linguistic characteristics	——
Qmark	Question marks	General description	——
RateSixLtrWord	Words>6 letters	General description	——
Relative	Relativity	Personal	area, bend, exit, stop
Religion	Religion	Personal	altar, church, mosque
See	See	Psychological characteristics	view, saw, seen
Social	Social process	Psychological characteristics	mate, talk, they, child
Space	Space	Personal	down, in, thin
TenseM	Tense marks	Linguistic characteristics	——
Tentat	Tentative	Psychological characteristics	maybe, perhaps, guess
Time	Time	Personal	end, until, season
Verb	Common verbs	Linguistic characteristics	walk, went, see
WordPerSentence	Words per sentence	General Description	——
Work	Work	Personal	job, majors, xerox

## Footnotes

^1^The Chinese term “JiaoZhen” means “truth-seeking.” The URL of Tencent “JiaoZhen” platform: https://vp.fact.qq.com/.

^2^The website URL of the COVID-19 pandemic situation disclosed daily by the National Health Commission of China is http://www.nhc.gov.cn/xcs/yqtb/list_gzbd.shtml.

^3^The introduction of LIWC and the definition of the dictionary can be found on its official website: http://liwc.wpengine.com/.
